# Maladaptive Plasticity and Neuropathic Pain

**DOI:** 10.1155/2016/4842159

**Published:** 2016-01-27

**Authors:** Xiang-Yao Li, You Wan, Shao-Jun Tang, Yun Guan, Feng Wei, Daqing Ma

**Affiliations:** ^1^Institute of Neuroscience and Collaborative Innovation Center for Brain Science, School of Medicine, Zhejiang University, Hangzhou, Zhejiang 310058, China; ^2^Department of Neurobiology, School of Basic Medical Sciences, Peking University Health Science Center, Beijing 100191, China; ^3^Department of Neuroscience and Cell Biology, University of Texas Medical Branch, Galveston, TX 77555, USA; ^4^Department of Anesthesiology and Critical Care Medicine, School of Medicine, Johns Hopkins University, Baltimore, MD 21205, USA; ^5^Department of Neural and Pain Sciences, Dental School, Program in Neuroscience, University of Maryland, 650 W. Baltimore Street, Baltimore, MD 21201, USA; ^6^Anaesthetics, Pain Medicine and Intensive Care, Department of Surgery and Cancer, Faculty of Medicine, Imperial College London, Chelsea & Westminster Hospital, London SW10 9NH, UK

The neuropathic pain is caused by the injury or damage on the sensory nervous system; its pathological manifestations include allodynia, hyperalgesia, and spontaneous pain. About 4–8% of people in our society are suffering from this disease. The maladaptive plasticity refers to the plasticity in the nervous system that leads to a disruption of the function and may be considered as a disease state [1]. Emerging evidences from both human patients and animal models showed that maladaptive plastic changes happened along the sensory pathways, from the peripheral to central nervous system, which may contribute to the generation, development, and maintenance of neuropathic pain. The current special issue focused on the molecular mechanism underlying maladaptive plasticity along sensory pathways; different laboratories around the world investigated the maladaptive change in different signaling pathways at different levels; the contributions in this area will highlight the pain managements in the future.

The transient receptor potential cation channel subfamily V member 1 (TRPV1) is the first identified member of TRPV proteins. TRPV1 can be activated by high temperature or specific chemical stimuli. The activation of TRPV1 in the terminal of nociceptive primary afferents conveys noxious information to the spinal cord and superspinal regions. Recent studies also identified critical roles of TRPV1 in the central regions of pain pathway in pain regulation. In the current issue, T. Jian et al. report that TRPV1 in dorsal root ganglia (DRG) modulates histamine H4 receptor-mediated itch. This finding will extend our understanding of the mechanisms of histamine-induced itch and the interaction of the itch and pain. S.-I. Choi et al. review recent studies on TRPV1-regulated pain, especially pathological pain, and suggest that TRPV1 contributes to pain maintenance. The new data may have revealed novel insights into the roles of spinal cord TRPV1 in regulating spinal plasticity, which may identify a new target for modulating pathological pain.

Low back pain affects a large number of patients. Deformation of DRGs and their nerve roots is implicated in the pathogenesis. W.-J. Han et al. evaluate the analgesic effects of a novel synthesized nitronyl (NIT) nitroxide radicals on radicular low back pain in a rat model generated by chronic DRG compression. The authors report a significant analgesic effect of this agent, probably due to its antioxidation and anti-inflammation effects. Y-.B. Xie et al. describe a new rat animal model of human low back pain, by chronic compression of multiple DRGs on one side of lumber. This rat model develops spontaneous pain, cold allodynia, and hyperalgesia. Activating transcription factor 3 (ATF3) and CGRP are upregulated in bilateral DRG neurons and may contribute to the expression of DRG compression-induced pain.

Matrix metalloproteinases (MMPs) play important roles in nociception and allodynia. Q. Wang et al. report a critical role of Extracellular Matrix Metalloproteinase Inducer (EMMPRIN)/OX47, a key regulator of MMP activities, in neuropathic pain development. Their studies show that SNL leads to OX47 upregulation in ipsilateral DRG and that downregulation of OX47 attenuates SNL-induced mechanical allodynia.

Gate-control theory of pain proposed by Melzack and Wall (1965) explains the regulation of noxious information transmission in the spinal substantia gelatinosa. F. J. R. Peláez and S. Taniguchi have revisited this theory from the perspective of NMDA receptor-mediated synaptic plasticity and intrinsic plasticity, and the Melzack and Wall circuit was slightly modified by using strictly excitatory nociceptive afferences and was further tested at different pain conditions.

Spinal microglia play critical roles in the developments of chronic pain. A. Jurga et al. report the contribution of TLR2 and TLR4 to neuropathic pain in a chronic constriction injury (CCI) model. They describe a time-dependent upregulation of TLR2, TLR4, MyD88, and TRIF mRNA and protein. In addition, blockade of TLR2 and TLR4 impairs the expression of pain behaviors and opioid analgesia. These findings support TLR2 and TLR4 as putative targets for developing therapeutic approaches.

J. Wang et al. report the involvements of mammalian target of rapamycin (mTOR) in the RVM, a key relay region for the descending pathway, in regulation of neuropathic pain. They show that phosphorylated mTOR protein increases mainly in RVM serotonergic spinally projecting neurons in the spared nerve injury (SNI) model. Infusion of rapamycin decreases both excitatory synaptic transmission and intrinsic excitability of serotonergic neurons, which may underlie the analgesic effects. These findings suggest a novel mechanism by which mTOR inhibitor causes analgesia.

The anterior cingulate cortex (ACC) is a heterogeneous brain region and is involved in regulation of pain, emotion, and sex attraction. Z.-X. Zuo et al. report that nerve ligation leads to AChE upregulation in the ACC. They have tested the analgesic effects of huperzine A, an alkaloid isolated from a Chinese club-moss, on evoked pain and spontaneous pain, and reported a significant analgesic effect on evoked pain but not spontaneous pain, indicating a specific role of AChE in regulation of evoked pain.

We are pleased to introduce this special issue that focuses on the maladaptive plasticity in the pain pathway, in both PNS and CNS. It is becoming clear that pain-related maladaptive changes can occur at a cellular level such as alteration of synaptic transmission and at a molecular level such as the dysregulation of various signaling pathways. The studies collected in this issue will help elucidate the pathogenic mechanism of neuropathic pain and facilitate the development of novel strategies for the pain treatments.
